# Visual outcomes after bilateral trifocal diffractive intraocular lens implantation

**DOI:** 10.1186/s12886-015-0012-4

**Published:** 2015-03-14

**Authors:** Jesús Carballo-Alvarez, Jose M Vazquez-Molini, Juan C Sanz-Fernandez, Javier Garcia-Bella, Vicente Polo, Julián García-Feijoo, Jose M Martinez-de-la-Casa

**Affiliations:** 1 Facultad de Óptica y Optometría, Universidad Complutense de Madrid, Madrid, Spain; 2 Servicio de Oftalmologia, Hospital Clinico San Carlos; Departamento de Oftalmologia, Facultad de Medicina, Universidad Complutense de Madrid; and Instituto de Investigacion Sanitaria del Hospital Clinico San Carlos (IdISSC), Madrid, Spain; 3 Hospital Miguel Servet, Zaragoza, Spain

**Keywords:** Cataract surgery, Multifocal, Trifocal, Intraocular lens, Contrast sensitivity

## Abstract

**Background:**

In recent years new models of intraocular lenses are appearing on the market to reduce requirements for additional optical correction. The purpose of this study is to assess visual outcomes following bilateral cataract surgery and the implant of a FineVision® trifocal intraocular lens (IOL).

**Methods:**

Prospective, nonrandomized, observational study. Vision was assessed in 44 eyes of 22 patients (mean age 68.4 ± 5.5 years) before and 3 months after surgery. Aberrations were determined using the Topcon KR-1 W wave-front analyzer. LogMAR visual acuity was measured at distance (corrected distance visual acuity, CDVA 4 m), intermediate (distance corrected intermediate visual acuity, DCIVA 60 cm) and near (distance corrected near visual acuity, DCNVA 40 cm). The Pelli-Robson letter chart and the CSV-1000 test were used to estimate contrast sensitivity (CS). Defocus curve testing was performed in photopic and mesopic conditions. Adverse photic phenomena were assessed using the Halo v1.0 program.

**Results:**

Mean aberration values for a mesopic pupil diameter were: total HOA RMS: 0.41 ± 0.30 μm, coma: 0.32 ± 0.22 μm and spherical aberration: 0.21 ± 0.20 μm. Binocular logMAR measurements were: CDVA −0.05 ± 0.05, DCIVA 0.15 ± 0.10, and DCNVA 0.06 ± 0.10. Mean Pelli-Robson CS was 1.40 ± 0.14 log units. Mean CSV100 CS for the 4 frequencies examined (A: 3 cycles/degree (cpd), B: 6 cpd, C: 12 cpd, D: 18 cpd) were 1.64 ± 0.14, 1.77 ± 0.18, 1.44 ± 0.24 and 0.98 ± 0.24 log units, respectively. Significant differences were observed in defocus curves for photopic and mesopic conditions (p < 0.0001). A mean disturbance index of 0.28 ± 0.22 was obtained.

**Conclusions:**

Bilateral FineVision IOL implant achieved a full range of adequate vision, satisfactory contrast sensitivity, and a lack of significant adverse photic phenomena.

**Trial registration:**

Eudract Clinical Trials Registry Number: 2014-003266-2.

## Background

Intraocular lenses (IOL) used in modern cataract surgery have been designed to achieve a good quality of vision at near and intermediate distance as well as far.

The FineVision® trifocal diffractive IOL (Physiol, Liège, Belgium) combines two diffractive structures that are adjusted to offer +3.50 D addition for near vision and +1.75 D addition for intermediate vision (http://www.physiol.eu/en/multifocal-iol/finevision/). Its design is such that the loss of light energy characteristic of diffractive systems is reduced. This energy gain significantly improves intermediate vision while maintaining performance for far and near vision. The diffractive anterior surface of the IOL is entirely convoluted. By varying the height of the diffractive step, the amount of light distributed to near, intermediate and distant foci is adjusted according to pupil aperture. The optic of the lens is designed to allocate 43% of light energy to far vision, 28% to near vision, and 15% to intermediate vision for a 3 mm aperture. The remaining 14% of light energy is lost.

Most of the available data in the literature relate to visual outcomes following bifocal and trifocal IOL implantation [1-3]. Few studies have assessed the FineVision IOL [4,5], especially in terms of aberrometry outcomes.

The present study was designed to determine visual and refractive outcomes in patients undergoing cataract surgery and the implant of a FineVision IOL in both eyes.

## Methods

This prospective experimental study adhered to the tenets of the Declaration of Helsinki. The study protocol was approved by the Hospital Clinico San Carlos review board and written informed consent was obtained from all patients.

To qualify for the study, it was required that patients had been diagnosed with cataract in both eyes, had no other ocular disease, and had not undergone prior ocular surgery. Subjects were included if they were 55 to 75 years old, had expressed a desire to be independent of spectacles and their pre-surgery refraction was a sphere of up to ± 5.00D and cylinder up to −1.50D.

All patients had cataract surgery by the same experienced surgeon (JMC) under topical anesthesia through a 1.8 mm clear corneal incision. Phacoemulsification was performed using the Stellaris system (Bausch & Lomb Incorporated, Rochester, NY) and this was followed by irrigation and aspiration of the cortex and IOL implantation in the capsular bag. The second eye operation was performed within 6 weeks of the first.

Three months after the second surgery, all patients underwent an optometric examination in which objective refraction and aberrometry were conducted using the wavefront analyzer Topcon KR-1 W (Oakland, USA). Corneal and internal aberrations were measured in a dark room in both photopic and mesopic conditions to induce physiologically normal pupil sizes.

Uncorrected visual acuity was determined monocularly (uncorrected distance visual acuity, UDVA) in photopic (85 cd/m^2^) luminance conditions using an ETDRS illumination cabinet with high contrast (96%) at a distance of 4 meters. Next, given that the IOLs in our patients were implanted in both eyes to optimize vision, distance (corrected distance visual acuity, CDVA 4 m), intermediate (distance corrected intermediate visual acuity, DCIVA 60 cm) and near (distance corrected near visual acuity, DCNVA 40 cm) visual acuity were measured binocularly using the EDTRS scale with distance correction under the same photopic conditions.

Monocular and binocular contrast sensitivity (CS) were first assessed using the Pelli-Robson letter chart (Clement Clarke International, Haag Streit UK, England) with best-spectacle correction and an addition of +1.00D at the same luminance level at 1 meter. Next, monocular and binocular CS were measured using the CSV-1000 test (Vector Vision, Greenville, OH) at 2.50 meters for 4 frequencies in cycles per degree (cpd) (A: 3 cpd, B: 6 cpd, C:12 cpd and D: 18 cpd).

Once distance correction had been established using the ETDRS chart at 4 meters, two additional lenses of the same power were simultaneously introduced in front of both eyes to produce defocus and then measure visual acuity. The range of lenses used was −4.00D to +1.50D in 0.50D steps. This method has been validated as a repeatable and reliable procedure to measure the amplitude of accommodation [6,7]. Given the FineVision IOL was designed to work in conjunction with pupil aperture, defocus curve testing was performed in both photopic and mesopic (3 cd/m^2^) conditions. Mesopic conditions were achieved using a filter that reduced the normal cabinet lighting level.

Adverse photic phenomena were assessed by halometry using Halo v1.0 software [8]. This program can be downloaded free from http://www.ugr.es/~labvisgr/. The test consists of discriminating peripheral luminous stimuli, 2 pixels in size, around a more luminous central target of 24 pixels. The central stimulus causes intraocular scattering and retinal reflection in the patient’s eye, depending on the state of the retina and ocular media. We examined 6 meridians of 60 degrees each. The discrimination capacity of peripheral stimuli in the presence of visual disturbances is evaluated by a parameter called the disturbance index. This index is calculated as a ratio between non detected stimuli and all the peripheral stimuli presented to the observer.

### Statistical analysis

Quantitative data are provided as ranges, means and standard deviations. The Student t-test for paired data was used to compare normally distributed data as confirmed using the Kolmogorov-Smirnov test, and the Wilcoxon rank-sum test was used for non-normally distributed data. All statistical tests were performed using the package SPSS Statistics v18.0. Significance was set at a p ≤ 0.05.

## Results

The final study sample was comprised of 44 eyes of 22 patients (10 men, 12 women). Mean age was 68.4 ± 5.5 years (range 55 to 75 years). Mean spherical refraction was −0.65 ± 2.10 D (range −4.75 to +3.50) preoperatively and 0.02 ± 0.44 D (range −1.00 to +0.50) postoperatively. Mean cylinder was −0.61 ± 0.67 D (range −1.50 to +1.00) preoperatively and −0.50 ± 0.35 D (range −1.00 to 0.00) postoperatively. Table 1 shows monocular UDVA and binocular CDVA, DCIVA and DCNVA for the study participants.

**Table 1:** **Monocular and binocular logMar visual acuity post IOL implantation**

	Monocular		Binocular	
	**Right eye**	**Left eye**	**CDVA**	−0.05 +/− 0.05
**UDVA**	0.23 +/−0.18	0.21+/− 0.12	**DCIVA**	0.15 +/− 0.10
**CDVA**	0.08 +/− 0.08	0.05+/− 0.07	**DCNVA**	0.06 +/− 0.10

*UDVA*: uncorrected distance visual acuity (4 m), *CDVA*: corrected distance visual acuity (4 m). *DCIVA*: distance**-**corrected intermediate VA (60 cm), *DCNVA*: distance-corrected near VA (40 cm).

**Figure 1:**
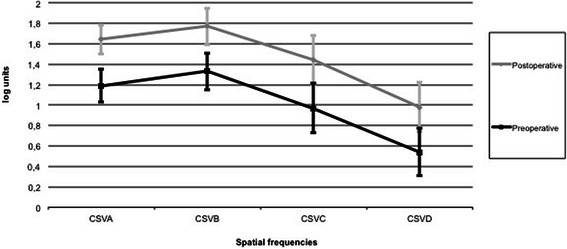
**Binocular contrast sensitivity functions pre and post IOL implantation under photopic conditions.**

Binocular CS using the Pelli Robson test showed a significant improvement after surgery (1.40 ± 0.14 vs 1.24 ± 0.18; p = 0.002). Figure 1 shows the pre and post surgery results of the CSV-1000 test under photopic (85 cd/m2) conditions for 4 spatial frequencies (A: 3 cpd, B: 6 cpd, C:12 cpd and D: 18 cpd). Post implantation mean binocular CS for the 4 frequencies examined (3, 6, 12 and 18 cpd) were 1.64 ± 0.14, 1.77 ± 0.18, 1.44 ± 0.24 and 0.98 ± 0.24 log units, respectively. In response to surgery, CS increases of 0.45, 0.44, 0.47 and 0.44 log units were produced for the 4 spatial frequencies, respectively (p < 0.0005). Further, monocular and binocular values significantly varied for all frequencies (p < 0.02).

**Figure 2:**
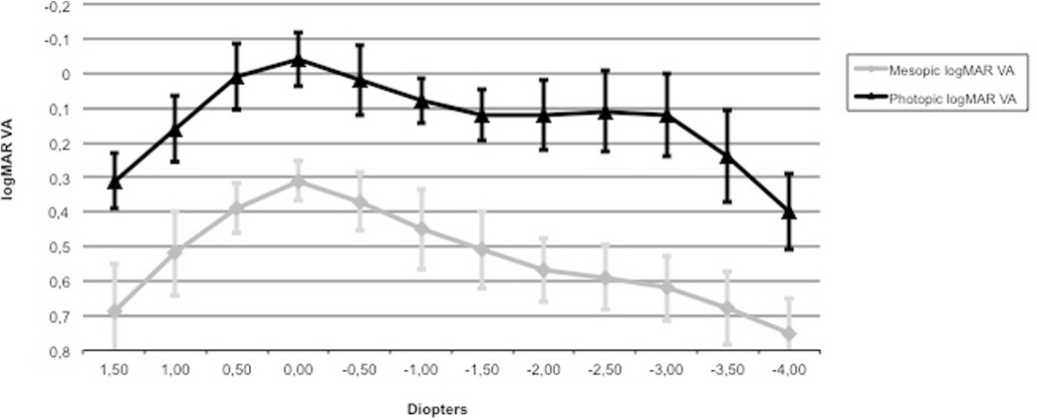
**FineVision binocular best distance-corrected defocus curve.**

Figure 2 shows postoperative through-focus corrected binocular logMAR visual acuity. The defocus curve consisted of one peak of maximum vision located at the far focus, corresponding to 0.00 D. Significant differences were detected between the far and intermediate (−1.50 D) focus (p < 0.0001) and between the far and near (−3.00 D) focus; p < 0.0001. No significant differences were found between the intermediate and near foci. A significant difference was observed between photopic and mesopic VA at far, intermediate and near distance (p < 0.0001).

**Figure 3:**
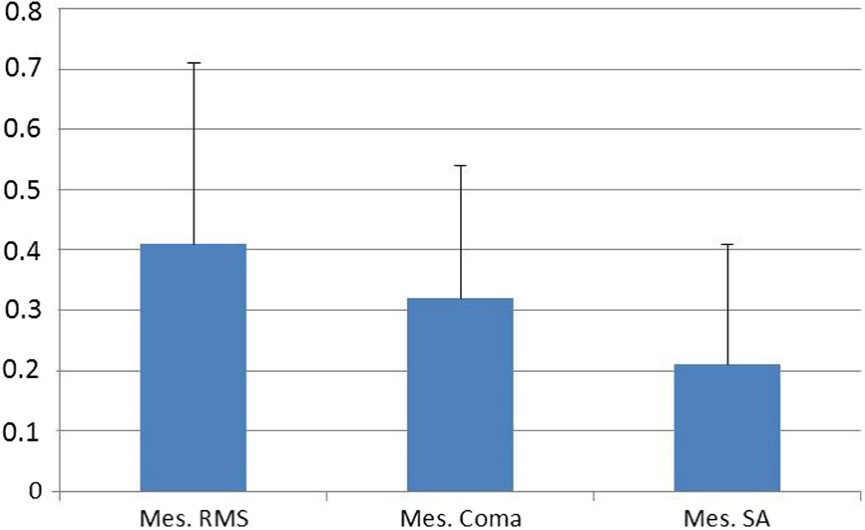
**HOA RMS(μm), coma and spherical aberration values at mesopic diameter.**

Figure 3 shows the aberrometry outcomes obtained after FineVision IOL implantation. Mean outcomes for a mean measured mesopic diameter of 4.67 ± 0.67 mm were: High Order RMS 0.41 ± 0.30 μm, coma 0.32 ± 0.22 μm, and SA 0.21 ± 0.20 μm.

The mean disturbance index for the 44 eyes was 0.29 ± 0.21 (range 0.00 to 0.70).

## Discussion

The FineVision trifocal diffractive IOL has three focal points for far, near and intermediate vision. This study examines the outcome of bilateral implantation of this IOL and provides far, intermediate and near visual acuities determined with best distance correction in photopic conditions, defocus curves for photopic and mesopic conditions, contrast sensitivity values assessed using two methods, along with halometry and aberrometry data.

Our findings indicate satisfactory visual acuity provided by the IOL at any distance both when tested subjectively using the visual acuity test and objectively by blurring vision with defocus addition lenses. Visual acuities remained satisfactory in photopic and mesopic conditions even under low contrast.

The early visual comfort observed shortly after surgery in our study indicates quick adaptation to the trifocal IOL examined. Alió [9] detected no differences in reading scores determined 1 and 6 months after the implant of a fully-diffractive lens, while scores for refractive and refractive-diffractive multifocal IOLs tended to improve over time. In particular, this author reported a significantly worse uncorrected reading speed in the refractive multifocal IOL group than in the monofocal IOL group at one month. Neuroadaptation to fully-diffractive IOLs is quicker, as pupil dynamics does not affect visual outcome.

Blaylock et al. [10] reported that after the implantation of a Restor +4D in 37 eyes (Alcon, USA), monocular distance visual acuity improved from logMAR 0.09 ± 0.10 without correction to logMAR 0.00 ± 0.05 with best correction. De Vries et al. [11] described uncorrected and best-corrected monocular visual acuity values of 0.04 ± 0.14 and −0.04 ± 0.09 after Restor +3D implantation (68 eyes) and of 0.14 ± 0.10 and −0.01 ± 0.06 after Restor +4D implantation (46 eyes), respectively. The best corrected visual acuity observed by de Vries et al. was much better than that observed by Blaylock et al. and that of the present study. This could be attributed to the significantly younger subjects examined by De Vries’ group. However, despite a similar visual acuity recorded for far vision, intermediate visual acuity derived from the monocular best distance-corrected defocus curve of de Vries et al. was lower than that reported in our study.

Owing to augmented spherical aberration, the contrast sensitivity anticipated for a multifocal diffractive IOL is lower than for healthy eyes. A loss of contrast sensitivity have been reported after the implantation of different models of multifocal IOLs related to the increase of HOA [12,13]. Previous studies showed that contrast sensitivity can increase for old people after surgery as a consequence of the elimination of the opacified crystalline lens. Although the aspheric surface contributes theorically to better optical quality and contrast sensitivity, especially under mesopic conditions, patients probably need longer follow up to reestablish the contrast sensitivity with some refractive multifocal IOL designs [13-15].

Our mean Pelli-Robson outcomes were similar to those described by Bautista et al. [16] using the same test for the multifocal IOL Tecnis ZMB00. Using the CSV-1000 test, we detected significant differences between monocular and binocular CS for all spatial frequencies and also between pre and postoperative CS values. In other studies examining diffractive IOL designs, binocular summation was also found to improve contrast sensitivity [5,17-19]. Consistent with the findings of Sheppard et al., our CS results were in the range expected for older subjects [5]. At lower luminance levels, CS would be likely reduced, as reported by Voskresenskaya et al. [3] for the MIOL-Record diffractive IOL.

Collectively these data suggest that trifocality does not induce a loss of contrast sensitivity compared to bifocal IOLs. This can be explained by the fact that Tecnis and FineVision allocate similar amounts of light energy to far vision. As far as we are aware, there are no other descriptions available of monocular visual acuity determined using the Pelli Robson test to assess IOL implantation outcomes.

The defocus curves of the studies by de Vries et al. [11] (monocular) for Acrysof Restor +4D and +3D, by Toto et al. [20] for Tecnis +4D, by Blaylock et al. [10] for Acrysof Restor +4D, and Alfonso et al. [2] (binocular) for Acrysof Restor + 4D, +3D, Acrilisa, all revealed a decrease in visual acuity (from logMAR 0.2 to 0.4) between −1 D and −1.5 D that we did not observe in our defocus curve. The decrease in visual acuity in the intermediate range of the defocus curve (from −1.00 to −2.00D) was no longer observed with the FineVision IOL. This gap is filled by the third focus with a +1.75 D addition at the IOL plane. Moreover, our mesopic and photopic defocus curves varied significantly, differences being more pronounced for intermediate and near vision. This is probably attributable to the lens design.

Aberrometry after multifocal IOL implantation is not totally reliable [21]. The amount of aberration detected here was comparable to that reported by Alio [22] for both Restor and Acrilisa IOLs for a 5.00 mm pupil diameter after cycloplegia. Ocular aberrations are highly pupil-dependent [14] and consequently our results obtained with different pupil diameters probably explains the large standard deviation.

Our binocular halometry study returned a better mean disturbance index than that reported by Castro et al. [8] for healthy eyes (mean index = 0.45 ± 0.10), but with a high standard deviation in our sample. In agreement with the findings of Cochener et al. [4] for the FineVision IOL, no patient reported photic phenomena. This is most likely attributable to increased far vision dominance as pupil size increases causing a reduction in the percentage of light reaching the rest of the foci. According to Voskrensakya et al. [3] and Souza et al. [23], complaints of halos and glare following the implant of a diffractive IOL tend to diminish over time.

## Conclusions

Distance visual outcomes of the trifocal IOL FineVision were similar to those of monofocal IOLs, while near visual outcomes resembled those of bifocal diffractive IOLs. In addition, a real improvement in intermediate vision was noted, which was not associated with impairment of far or near vision. Trifocality did not lead to increased subjective visual discomfort in comparison to bifocality. Moreover, the indications and limitations of the FineVision lens do not differ from those of other diffractive lenses.
